# Multifunctional Eco-Friendly Adsorbent Cryogels Based on Xylan Derived from Coffee Residues

**DOI:** 10.3390/membranes14050108

**Published:** 2024-05-08

**Authors:** Valentina Quintero, Johann F. Osma, Ulugbek Azimov, Debora Nabarlatz

**Affiliations:** 1 Grupo de Investigación en Tecnologías de Valorización de Residuos y Fuentes Agrícolas e Industriales para la Sustentabilidad Energética (INTERFASE), Escuela de Ingeniería Química, Universidad Industrial de Santander, Cra. 27 N°9, Bucaramanga 680002, Colombia; laura.quintero5@correo.uis.edu.co; 2 BioAgro Center, Innovation and Technology Inc., Guasca 251217, Colombia; faccelo@yahoo.com; 3 Faculty of Engineering and Environment, Northumbria University, Newcastle upon Tyne NE1 8ST, UK; ulugbek.azimov@northumbria.ac.uk

**Keywords:** xylan, coffee parchment, lignocellulosic-based materials, cryogel, ammonia capture, greenhouse gas emissions

## Abstract

Agricultural and animal farming practices contribute significantly to greenhouse gas (GHG) emissions such as NH_3_, CH_4_, CO_2_, and NO_x_, causing local environmental concerns involving health risks and water/air pollution. A growing need to capture these pollutants is leading to the development of new strategies, including the use of solid adsorbents. However, commonly used adsorbent materials often pose toxicity and negative long-term environmental effects. This study aimed to develop responsive eco-friendly cryogels using xylan extracted from coffee parchment, a typical residue from coffee production. The crosslinking in cryogels was accomplished by “freeze-thawing” and subsequent freeze-drying. Cryogels were characterized in terms of morphology by using scanning electron microscopy, porosity, and density by the liquid saturation method and also moisture adsorption and ammonia adsorption capacity. The analysis showed that the porosity in the cryogels remained around 0.62–0.42, while the apparent densities varied from 0.14 g/cm^3^ to 0.25 g/cm^3^. The moisture adsorption capacity was the highest at the highest relative humidity level (80%), reaching 0.25–0.43 g of water per gram of sample; the amount of water adsorbed increased when the xylan content in the cryogel increased up to 10% *w*/*v*, which was consistent with the hygroscopic nature of xylan. The ammonia adsorption process was modeled accurately by a pseudo-second-order equation, where the maximum adsorption capacity in equilibrium reached 0.047 mg NH_3_/g when xylan reached 10% *w*/*v* in cryogels, indicating a chemisorption process. The cryogels under investigation hold promise for ammonia adsorption applications and GHG separation, offering a sustainable alternative for gas-capturing processes.

## 1. Introduction

According to FAO 2020, agriculture and livestock are a major source of NH_3_, CH_4_, CO_2_, and NO_x_ emissions, where they represent 17% of total global greenhouse emissions. They are also responsible for local environmental problems, such as health problems, water and air pollution, and phosphorus and nitrogen management failures [1,2]. Among these pollutants, ammonia has significant negative environmental impacts on, for example, air, soil, and water quality, also contributing to the formation of particulate matter which affects human health [3]. Ammonia gas emissions have been regulated for several years already by the Gothenburg Protocol to Abate Acidification, Eutrophication, and Ground-Level Ozone (UNECE, 2018) and by the Kyoto Protocol arising from the UN Framework Convention on Climatic Change (UN, 1997), respectively.

For example, common levels of ammonia in poultry farms are typically measured in parts per million (ppm) and are often around 50 ppm or more, depending on the conditions of the place, where in poorly ventilated houses, ammonia concentration may reach 200 ppm [4]. In Colombia, the current regulation establishes that the permissible levels of air quality or emission in activities that generate offensive odors such as ammonia, should not exceed 2.01 ppm per hour and 0.13 ppm for an exposure period of 24 h [5].

The growing need to capture these pollutants is leading to the development of new strategies and approaches. Among them, the use of specific adsorbents is growing (e.g., zeolites impregnated with amines, MOFs, etc.), mainly due to these materials having a large surface area and large pore volume, and they are selectively functionalized for gas capture [6]. However, they are often toxic, have poor biodegradability, or have a complicated and costly fabrication process [7,8].

Over the past few decades, hydrogels have received increased interest due to their properties and functions as well as simple preparation methods and diversity of applications, ranging from tissue engineering, drug delivery systems, sensors, and even water treatment. Hydrogels are three-dimensional crosslinked polymeric networks, which can adsorb and retain large amounts of water [7]. The process of the formation of hydrogels is carried out by the physical or chemical crosslinking of the polymeric chains. In physical crosslinking, the molecular interactions can be either strong or weak and may be given by hydrogen bonds, electrostatic interaction, hydrophobic interaction, crystallization, etc. In the case of chemical crosslinking, these interactions are given by covalent bonds, free radical polymerization, etc.; those kinds of interactions are much stronger than physical ones [9]. Simple and low-cost methods of hydrogel preparation such as freeze-thawing allows some polymers such as polyvinyl alcohol (PVA) to physically crosslink, achieving gelation. This method consists of mixing the precursor materials and subjecting the mixture to several freeze-thawing cycles [7,10].

According to the final application of the hydrogel, there are different ways to prepare hydrogels to be used as porous adsorbent materials. One of these methods is drying them to remove the solvent; however, conventional drying ways, such as evaporation, produce the collapse of the pores due to high capillary pressure, leading to denser and lower porosity materials (xerogels) [11]. On the other hand, freeze-drying and supercritical drying preserve the porous structure of the hydrogel better, whereas freeze-drying allows the formation of large pores and ten-micron-sized channels due to the growth of ice crystals which are then sublimated (cryogels). During supercritical drying, there is no surface tension preserving the structural network (aerogels) [11,12].

One widely used material to produce hydrogels is polyvinyl alcohol (PVA), which is a synthetic material with some attractive properties such as water solubility, biodegradability, non-toxicity, and good chemical and mechanical resistance. For the formation of hydrogel and crosslinking of PVA, techniques such as freeze-thawing were used, so that PVA physically crosslinked with other polymeric chains [13,14,15]

Several studies have reported its use in blends with polysaccharides and natural polymers such as starch, gelatin, collagen, chitosan, and dextran, among others [10,13,16]. Xylan is the most common form of hemicellulose found in lignocellulosic residues (25–30%), which is itself a type of “heteropolymer”, consisting of sugars composed of pentoses and hexoses. Its structure is commonly composed of β-(1,4)-xylose linkages, with side chains consisting mainly of α-d-glucuronic acid, 4-*O*-methyl-α-d-glucuronic acid, different sugar units (α-l-arabinofuranose, α-d-xylopyranose or α-d-galactopyranose), and several substituents such as acetyl, phenolic, and ferulic groups [17,18]. Hemicellulose, especially xylan, has been used as a polymer for the formulation of gels and thermoplastic materials [19,20]. Xylan has received special attention for the preparation of biosorbents used as a drug release agent, biomaterial for osteonecrosis treatment and conductive material, and hydrogel formulation effective for the adsorption of heavy metals such as Pd^2+^, Cd^2+^, and Zn^2+^ [15].

As mentioned, xylan can be obtained from different hemicellulosic sources, including agro-industrial wastes. In this sense, agricultural activity plays a significant role in the economy and development of Colombia. Agro-industrial crops, such as coffee, palm oil, sugar cane, cocoa, soybeans, and cotton, account for 41.2% of the total area planted in Colombia [21]. For example, of the annual production of coffee in Colombia, it is estimated that 2,849,596 tons of waste are generated and less than 5% is used, representing an opportunity for biomaterials production and other applications [22,23].

In the present work, eco-friendly adsorbent cryogels were synthesized using xylan extracted from coffee parchments. The cryogels were prepared by varying the xylan ratio in the mixture, while the crosslinking was carried out by freeze-thawing followed by freeze-drying. Cryogels were then characterized to study their morphological structure, water adsorption capacity, and ammonia gas adsorption capacity for their use as greenhouse gas adsorbents.

## 2. Materials and Methods

### 2.1. Materials

The coffee parchment was provided by local farmers in San Gil, Santander, Colombia. Partially hydrolyzed polyvinyl alcohol (PVA) was purchased from SUQUIN (Bucaramanga, Colombia). Bentonite was purchased from Biocombustibles Sostenibles del Caribe S.A (Santa Marta, Colombia). Alginate was purchased from Jinan Boss Chemical Industry Co. (Jinan, China). Ethanol (99% purity) was purchased from JT Baker (Fisher Scientific, Thermo Fisher Scientific, Waltham, MA, USA). Ammonium chloride (NH_4_Cl, purity 99.9% for analysis) was purchased from Merck (Merck KGaA, Darmstadt, Germany). Acetonitrile was purchased from Merck (Merck KGaA, Darmstadt, Germany). Glucose, xylose, arabinose, and dextran standards (6, 20 and 40 kDa) were purchased from Alfa Aesar (Haverhill, MA, USA). Distilled water was used for all the experiments. Ultrapure water was used for chromatography analysis.

### 2.2. Xylan Extraction from Coffee Parchment

The coffee parchment biomass used as feedstock in this work was characterized and had the following average composition (in dry mass basis): 48.7 ± 0.75 wt.% cellulose, 22.8 ± 1.22 wt.% hemicellulose, 18.8 ± 0.91 wt.% klason lignin according to standard norm [24] ASTM D1103-60 and ASTM D1106-96 [25], respectively), 0.88 ± 0.13 wt.% ash according to standard norm ASTM D7582-10 [26], 4.04 ± 0.15 wt.% aqueous extractives, and 0.09 ± 0.08 wt.% organic extractives according to standard norm ASTM D1110-84 and ASTM D1105-21 [27,28]. Xylan was obtained by the autohydrolysis of coffee parchments according to Acosta-Fernandez et al (2018) [29]. Autohydrolysis was carried out in a 20 L batch reactor at 160 °C using 1:8 (*w*/*v*) biomass/water ratio during 2 h. After stopping the reaction, the liquor containing the dissolved xylan was filtered and separated from the remaining solid fraction and then frozen in storage at −20 °C. The liquor was then freeze-dried at −42 °C and −0.1 bar.

The composition of the freeze-dried powder (xylan) was determined by HPLC. To this purpose, 0.2 g of the lyophilized powder (xylan) was dissolved in 20 mL of distilled water under stirring. From this solution, a sample of 2 mL was filtered through a 0.22 μm syringe filter and analyzed by HPLC to quantify monosaccharides. Another sample of 5 mL was taken and mixed with 1 mL of 5N H_2_SO_4_, as was reported previously by Nabarlatz, Farriol, and Montané (2005) [30] for a post-hydrolysis reaction. The acidified solution was then hydrolyzed at 120 °C for 45 min to convert all oligosaccharides into their constitutive monomers, determining the oligomers present in liquid phase by the difference between the total monomers and hydrolyzed monomers. Finally, the solution was then filtered through a 0.22 μm syringe filter, and the monosaccharides were quantified by HPLC.

The HPLC analysis for the quantification of the main carbohydrates (glucose, xylose, and arabinose) was carried out in a Thermo Dionex Chromeleon chromatograph (Thermo Fisher Scientific, Waltham, MA, USA) equipped with a refractive index (RID) and ultraviolet (UV) detectors. The method used a Zorbax Carbohydrate column at 30 °C, with a mobile phase of acetonitrile/water (70:30) *v*/*v* at a flow rate of 1.2 mL/min. The xylan molecular weight was also determined by SEC with a Sepax column at 30 °C using acetonitrile/water (10:90) *v*/*v* at a 0.3 mL/min flow rate as the mobile phase.

### 2.3. Preparation of Cryogels

The cryogels were prepared according to the methodology described by Fathi et al. (2011) [7] and Dong et al. (2006) [31]. The solution was prepared by dissolving 0.1 g alginate, 0.5 g bentonite, and the lyophilized xylan in different percentages in 10 mL of distilled water. The solution was then sonicated in an ultrasonic bath for 1 h. After this, 10 mL of PVA at 20% *w*/*v* solution was added to this mixture. The final solution was composed by 0.5% *w*/*v* alginate, 2.5% *w*/*v* bentonite, 10% *w*/*v* PVA, and lyophilized xylan in different percentages, comprising 1, 3, 5, 7, and 10% *w*/*v*. The samples were maintained in the autoclave for 2 h at 120 °C and 1.5 bar pressure, thus completely dissolving the mixture. The solution was then cooled at room temperature (22.5 °C), poured in a Petri dish, and subsequently frozen at −20 °C for 20 h. After this first freezing, the samples were held and allowed to thaw at room temperature (for 4 h). This process of the freeze-thawing cycles was repeated 4 times according to [7] to develop mechanically acceptable hydrogels for future experiments. Finally, the hydrogels were freeze-dried (at −42 °C, −0.1 bar) for 48 h.

### 2.4. Characterization of Cryogels

#### 2.4.1. Porosity Measurements

The porosity of the cryogels was measured according to Zu et al. (2012) [32] by the liquid displacement method. To this purpose, 1 g of the cryogel was placed in a 20 mL beaker and weighted (W_1_). Then, an inert solvent (ethanol) was poured in until completing 20 mL, measuring W_2_. The cryogel was then removed and the beaker weighed again (W_3_). The cryogel pores were full of ethanol and the volume of ethanol in the pores was taken as the pore volume of the cryogel. The porosity (P) was calculated using the following equations:

(1) $$V_{C}=20-\left(\frac{W_{2}\;-{\;W}_{1}\;-\;1}{\rho_{E}}\right)$$

(2) $$\rho_{C}=\frac{1}{V_{C}}$$

(3) $$V_{E}=\frac{W_{2}\;-{\;W}_{3}\;-\;1}{\rho_{E}}$$

(4) $$P=\frac{V_{E}}{V_{E}+{\;V}_{C}}$$

where ρ_C_ is the density of the cryogel (g/cm^3^), ρ_E_ is the density of the ethanol (g/cm^3^), V_C_ is the volume of the cryogel (cm^3^), V_E_ is the volume of the ethanol in the pore (cm^3^), and P is the porosity (% *v*/*v*).

#### 2.4.2. Moisture and Hygroscopic Sorption Capacity

To determine the moisture of the cryogels and the hygroscopic sorption capacity, they were analyzed using the standard methods ASTM D7582-10 (moisture content) and ASTM C1498 (hygroscopic sorption isotherm) by triplicate [26,33].

#### 2.4.3. SEM, EDS, and BET Area Analysis

The cryogels were mounted onto metal stubs using carbon adhesive tape and a sputter coated with gold. The coated samples were observed using a QUANTA FEG (Field Emission Gun) 650 scanning electron microscope (Quanta System S.P.A., Milan, Italy), at the required magnification. EDS (energy dispersive spectroscopy) microanalysis was performed by using an EDAX APOLO X detector (EDAX, Warrendale, PA, USA), with 126.1 eV (en. Mn Kα) resolution in order to obtain a semi-quantitative elemental composition of the materials.

The BET area analysis was performed in 3A Flex Micromeritics (Micromeritics, Norcross, GA, USA). The samples were weighed in 9 mm diameter cells made of borosilicate glass (Micromeritics). They were degassed at 150 °C and 6 Pa for 12 h in a Vac Prep 061 (Micromeritics). The capture of the nitrogen and adsorption isotherms was performed at 77 K in a 3FLEXTM Micromeritics instrument (Micromeritics, Norcross, GA, USA) in a relative pressure range (P/P0) between 0.0025 and 0.9999. The data analysis was performed with the 3FLEX V.4.03 program provided by the equipment, and the surface areas ABET (m^2^/g) and pore volume (cm^3^/g) were determined.

#### 2.4.4. Ammonia Adsorption Capacity

A commercial MQ-137 sensor was used to detect the ammonia concentration during the batch adsorption process. This sensor is an electronic device that was programmed on the open-source electronics platform Arduino^®^ IDE 1.8.19 (Arduino, Somerville, USA) and is composed of the MQ-137 ammonia detection sensor, connected to the Arduino UNO^®^ Board (Dynamo Electronics S.A.S, Bucaramanga, Colombia). The sensitive material of the sensor is metal oxide (SnO_2_). The change in ammonia concentration causes a change in electrical resistance, which is measured and used for ammonia concentration estimations. The plate allows the reading of both as an analog value and as a digital value when a certain threshold is exceeded and regulated through a potentiometer located on the plate.

**Figure 1:**
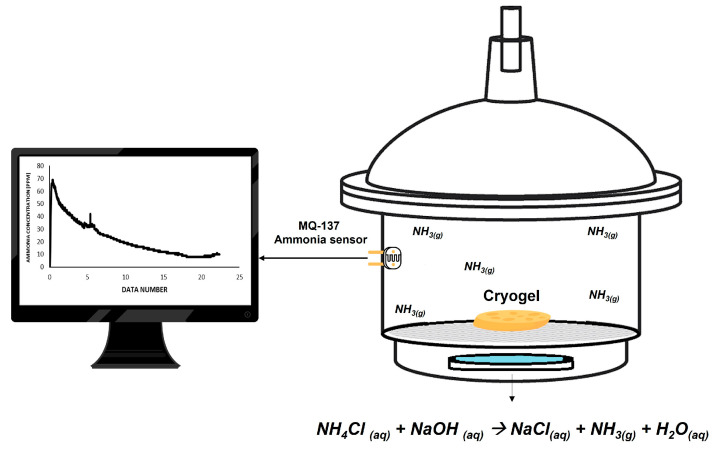
Set-up used for monitoring ammonia capture by MQ-137 sensor via Arduino^®^ and MATLAB^®^.

In order to monitor the ammonia adsorption capacity of the cryogels in a controlled environment, a set-up apparatus was built (see Figure 1). It consisted of a sealed desiccator (9.2 L volume) including a mesh used as support and equipped with the MQ-137 ammonia sensor connected to a data logger and a computer to register the data of ammonia concentration in the headspace of the desiccator (in ppm_v_). Communication was established with a serial port object, which was created in the MATLAB^®^ version R2022b App designer workspace.

To have ammonia concentrations according to the levels that are present in environments such as poultry houses or pig farms, ammonia was generated in situ through a reaction between ammonium chloride and sodium hydroxide, as following Equation (5):

NH_4_Cl_(aq)_ + NaOH_(aq)_ → NaCl_(aq)_ + NH_3(g)_ + H_2_O_(aq)_ (5)

The solution was prepared by adding 0.01 g of NH_4_Cl and 0.15 g of NaOH to 20 mL of distilled water under manual stirring (25 °C). The solution was then placed in the set-up, being closed and monitored for 24 h for detecting the ammonia formation and stabilization of the sensor. The ammonia concentration was monitored by a MQ-137 sensor (Dynamo Electronics S.A.S, Bucaramanga, Colombia) and reported in ppm_v_.

After that, the set-up was open, a new solution was added, and the sample was located in the support. The adsorption of ammonia was then monitored for 24 h. The concentration of ammonia detected in both cases (without and with cryogel) was calculated as follows:

(6) $$m_{{\mathrm{NH}}_{3}}\left(\mathrm{mg}\right)=\frac{({\mathrm{ppm}\;}_{v\;NH_{3}})*\frac{V_{T}\left(L\right)}{1000000}}{0.08206\;\left(L*\frac{\mathrm{atm}}{K*\mathrm{mol}}\right)*297\;\left(K\right)}*{\mathrm{MW}}_{{\mathrm{NH}}_{3}}(\frac{g}{\mathrm{mol}})*1000$$

where $V_{T}=9.2$ L and ${\mathrm{MW}}_{{\mathrm{NH}}_{3}}$ corresponds to the molecular weight of ammonia (17 g mol^−1^). The ammonia adsorption capacity (q_t_), in mg NH_3_ per gram of cryogel, as function of time was calculated as follows:

(7) $$q\;(t)=\frac{\left(C_{0}\;-{\;C}_{t}\right)\;V}{w}$$

where w is the amount of the adsorbent (in grams) used for the experiment, V is the volume of available gas space (9.2 L), C_0_ is the initial concentration of ammonia without the adsorbent (in ppm_v_), and C_t_ is the concentration of ammonia in the presence of cryogels (in ppm_v_) over time t (in seconds). Multiplying the adsorbate concentration by the volume gives

(8) $$q(t)=\frac{m_{0\;{\mathrm{NH}}_{3}}(\mathrm{mg})\;-\;m_{\left(t\right)NH_{3}}\;(\mathrm{mg})}{w\;(g)}$$

where w is the amount of the adsorbent (in grams) used in the experiments, $m_{0{NH}_{3}}$ is the mass of the initial ammonia when no cryogel is present (in mg) in the headspace, and $m_{(t)NH_{3}}$ is the mass of ammonia in the headspace with the presence of cryogel (in mg) at time t.

To evaluate the kinetics of the adsorption process, the kinetic data were correlated with pseudo-first-order and pseudo-second-order kinetic models. Correlation coefficient R^2^ was considered as a criterion to evaluate the correlated data [34]. Lagergren’s pseudo-first-order kinetic model is described as [35]

(9) $$\frac{d\;q(t)}{\mathrm{dt}}=k_{1}\left[q_{e}-\;q\left(t\right)\right]$$

where q_e_ and q(t) (mg NH_3_ g_sample_^−1^) are the adsorption removal capacities at equilibrium and variable time (t), respectively. K_1_ is the rate constant for the pseudo-first-order kinetic model (mg NH_3_ g_sample_^−1^ s^−1^). The Equation (9) can be integrated in the limits q(t) = 0 at t = 0 and q(t) = q(t) at time t = t to the following form:

(10) $$q\left(t\right)=q_{e}(1-e^{{-k}_{1}t})$$

Equation (12) is commonly used in the linearized form developed by [36]:

(11) $$\ln\left[q_{e}-\;q\left(t\right)\right]=-k_{1}t+\ln q_{e}$$

When the condition q_e_ ≪ k_1_/k_2_ is not satisfied, the second-order rate expression, commonly known as the pseudo-second-order kinetic model expression, can be used, which after integration, is formulated as follows:

(12) $$q\left(t\right)=\frac{q_{e}^{2}k_{2}t}{1+q_{e}k_{2}t}$$

where q_e_ and q_t_ (mg NH_3_/g_sample_) are the adsorption removal capacities at equilibrium and variable time (t), respectively. K_2_ is the rate constant for the pseudo-second-order kinetic model (g_sample_ s mg^−1^). Equation (13) is used in its linear form developed by [36]:

(13) $$\frac{t}{q(t)}=\left(\frac{1}{q_{e}}\right)t+\frac{1}{k_{2}q_{e}^{2}}$$

## 3. Results and Discussion

### 3.1. Xylan Extraction from Coffee Residues

**Figure 2:**
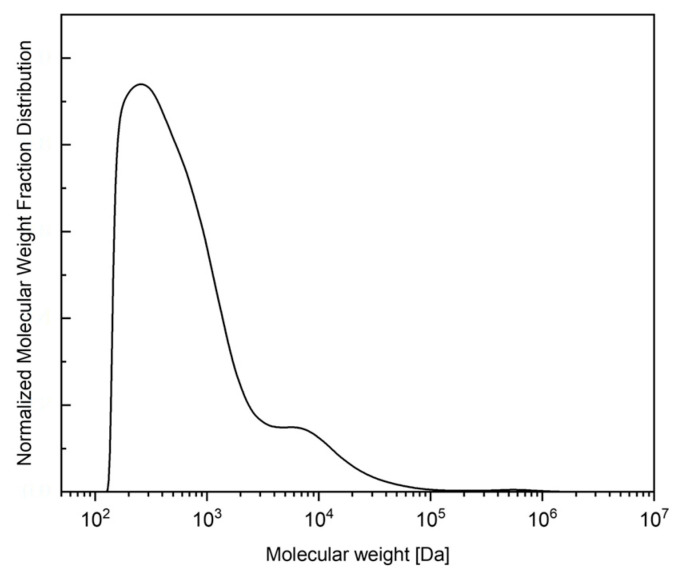
Normalized molecular weight distribution of the freeze-dried autohydrolysis liquor.

Xylose and arabinose as oligomers in the freeze-dried powder were 0.248 ± 0.016 [g xylose and arabinose/g freeze-dried powder]. The molecular weight distribution in the freeze-dried powder was 100–10,000 Da, as shown in Figure 2, where the xylan recovered had a similar molecular weight distribution to those produced by Acosta (2019) [37] from coffee parchment, where 69% of the sample had a molecular weight higher than 3 kDa. In previous research [38], it was mentioned that the molecular weight of xylan can have an important influence on the behavior and properties of the hydrogels obtained. While polymers with too-high molecular weights (>18 kDa) can result in a drastically decrease in their solubility, polymers with molecular weights lower than 2 kDa can affect the gelation properties during hydrogel formation.

### 3.2. Cryogels Characterization: Density and Porosity Analysis

**Figure 3:**
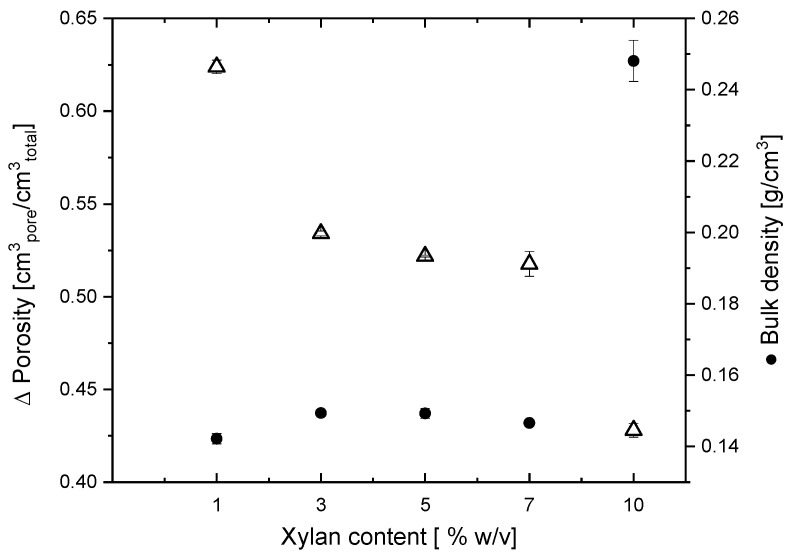
Porosity (Δ) and bulk density (•) of cryogels as function of xylan content. Error bar represents standard deviation.

The effect of the increase in the xylan proportion in the cryogel preparation on the density and porosity of the cryogels is shown in Figure 3. As the xylan content increases, the porosity of the cryogel decreases from 0.62 to 0.42 cm^3^ _pore_/cm^3^ _total_, indicating that cryogels are porous, which favors water and pollutant adsorption through capillary action [32]. These results are in agreement with those from Appelo (2013) [39], where the interlayer porosity due to bentonite can be affected by the addition of some materials (e.g., an added heteropolymer such as xylan) due to the osmotic effect. On the contrary, the bulk density of the cryogels varied from 0.14 to 0.24 g/cm^3^, in concordance with the results reported for siliceous materials used for adsorption where the density values are in the range between 0.17 and 0.45 g/cm^3^ [40]. When the xylan content in the cryogel reaches 10% *w*/*v*, the xylan dry mass in this hydrogel accounts for approximately 40 wt% of the material. As the xylan proportion in the material increases, the hygroscopicity of the material increases due to the fact that xylan incorporates hydroxyl groups which can form hydrogen bonds with other polymers, leading to the formation of a more densely packed structure, lowering porosity, increasing density, and therefore making the material suitable for capturing hydrophilic compounds [41].

### 3.3. Cryogels Characterization: Hygroscopic Sorption Capacity

**Figure 4:**
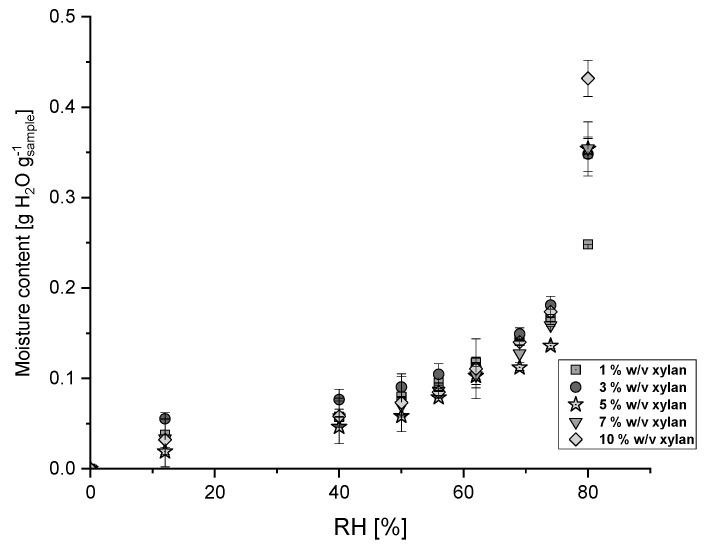
Moisture content of cryogels as a function of relative humidity (% RH) and xylan content.

The values of equilibrium moisture content in cryogels at various levels of relative humidity (RH) at 24 °C are shown in Figure 4. According to N. A. Aviara (2020) and Andrade P. et al. (2011) [42,43], type II adsorption profiles were obtained, being characteristic of hygroscopic products, considering the existence of an internal multilayer surface in the material. Cryogels seem to have a stable sorption behavior at low relative humidity values, and due to the RH lower than 40%, cryogels adsorb less than 0.1 g water per g of the sample. However, when the relative humidity increases above 50%, they become more hygroscopic, increasing the water adsorption until near 0.4 g of water per gram of the sample. At the highest RH level (80%), the amount of water adsorbed accounts to 0.43 g of water per gram of the sample increases when the xylan content in the cryogel also increases (10% *w*/*v* xylan), which is consistent with the hygroscopic nature of xylan. The moisture adsorption capacity can be related to the ammonia adsorption capacity, although not directly nor proportionally [44].

### 3.4. Morphology Characterization by SEM

**Figure 5:**
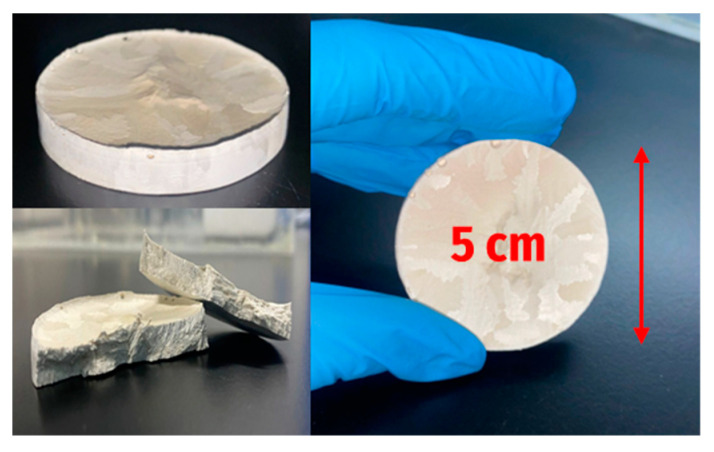
Visual aspect of cryogels obtained by freeze-thawing.

**Figure 6:**
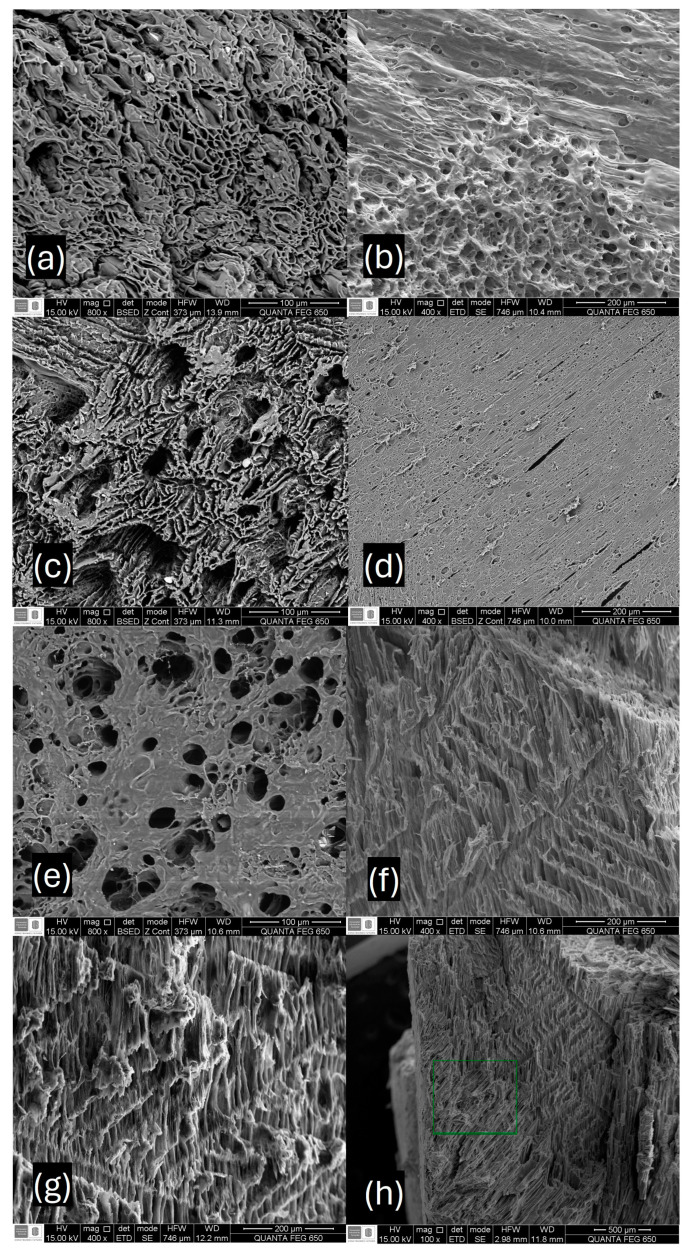
Surface SEM images of microstructure of cryogels: (**a**) xylan 1% *w*/*v*; (**b**) xylan 3% *w*/*v*; (**c**) xylan 5% *w*/*v*; (**d**) xylan 7% *w*/*v*; (**e**) xylan 10% *w*/*v*; (**f**–**h**) cross-cutting section SEM images of cryogels microstructure of cryogels with 1% *w*/*v*, 3% *w*/*v,* and 5% *w*/*v*.

The physical visualization of the cryogels obtained is shown in Figure 5. They are light structures, with a spongy appearance similar to expanded polystyrene. Figure 6 depicts the SEM micrographs of cryogels. As can be observed, cryogels are porous, where the porosity is formed apparently by the crystallization of water during the freeze-thawing process, and subsequent freeze-drying [7]. The porous surface has a high pore overlap, as well as large and interconnected sites. Cryogel with 1% *w*/*v* of xylan is observed in Figure 6a, where its surface presents an open and interconnected porosity like a “reef”-type structure. The pore diameter of the surface studied is 9.23 μm, suggesting a microporous structure. Figure 6b,c shows 3% and 5% *w*/*v* xylan cryogel, where the porous structure on the surface changes compared with the previous one, showing regular sized pores in some areas beside other denser areas which lack open porosity. The pore diameters, in this case, are 20.13 μm and 15.56 μm, respectively, suggesting that the increase in the amount of xylan contributes to the formation of more closed and dense structures.

This effect is most notorious when the xylan content increases to 7% and 10% *w*/*v*. For the cryogel with 7% *w*/*v* xylan, the average pore diameter in the open zones decreases to 13.73 μm, while for the sample with higher xylan content (10% *w*/*v*, Figure 6e), it presents a more uniform structure over the entire surface of the material, showing a pore diameter of 15.08 μm.

The elemental composition analysis performed by SEM-EDS showed the main composition of 71.11% ± 0.04 carbon (C) and 26.94% ± 0.04 oxygen (O) for all the samples. Other elements were detected in smaller quantities (<3%) such as sodium, aluminum, silica, magnesium, and potassium, which were associated with the composition of the bentonite added to the material, suggesting that adding bentonite to the cryogel did not affect substantially the sample’s composition, and the cryogels were mostly organic.

In Figure 6f–h, the cross section of the samples can also be observed for cryogels with 1% *w*/*v*, 3% *w*/*v*, and 5% *w*/*v* of xylan, respectively. It is possible to notice an aligned microstructure pattern in some areas formed by small fibrils. This type of microstructure was reported previously and was observed in polyvinylalcohol (PVA) xerogels, where this phenomenon was called transverse isotropic freeze-drying [45], these structures being caused due to the crosslinking of PVA when subjected to drastic changes in temperature and pressure. However, no aligned microstructures were formed in cryogels with higher amounts of xylan (7 and 10% *w*/*v*).

### 3.5. Brunauer–Emmett–Teller (BET) Analysis

Table 1 presents the results of the analysis of cryogels with 1% *w*/*v* xylan, 5% *w*/*v* xylan, and 10% *w*/*v* xylan. The results show that the addition of xylan reduces the porosity, being in agreement with previous reports where it was mentioned that the development of porous biomaterials is affected by factors such as temperature, humidity, and the solvents used; therefore, a low temperature and high water content can lead to a smaller pore size [46]. In general, biopolymers vary in surface area and pore volume depending on their structure and manufacturing processes [47]. However, these results are comparable to the BET surface area reported for activated carbons prepared from bamboo (carbonized at 400 °C without any acid treatment), reaching values of 2.17 m^2^/g and a total pore volume lower than 0.001 cm^3^/g, which were used also to study ammonia adsorption [48].

### 3.6. Cryogels Ammonia Adsorption Capacity

**Figure 7:**
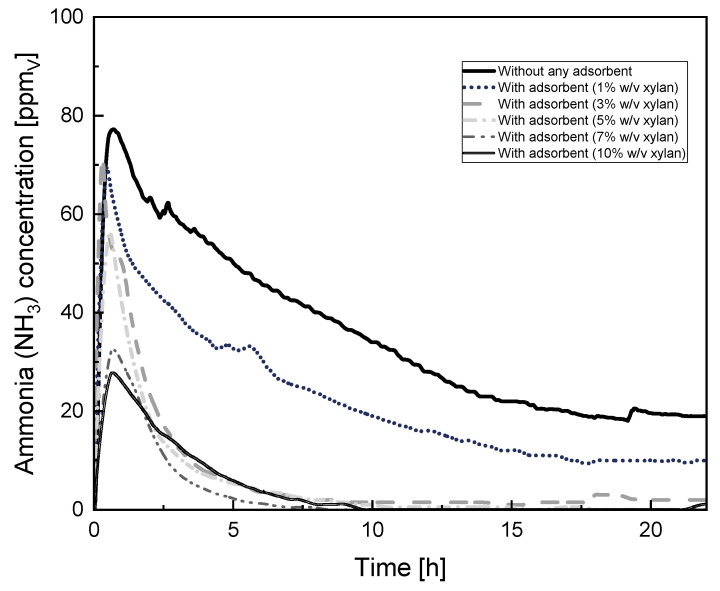
Capture of ammonia in cryogels as a function of xylan content and time.

The ammonia adsorption of the cryogels as a function of the percentage of xylan and time is shown in Figure 7. The continuous curve represents the ammonia concentration detected by the sensor in the container when no adsorbent is present, demonstrating the natural reaction of ammonium chloride and sodium hydroxide forming gas ammonia and filling the space by natural convection. Equilibrium in the environment was reached at approximately 15 h, where the ammonia concentration stabilized at around 19 ppm_v_. When the cryogel was placed and the container was closed, cryogels started adsorbing ammonia from the environment. An enhancement in adsorption of ammonia was observed with the increase in the xylan content in the cryogels, represented by the dotted lines. These results demonstrated that the increase in the adsorption capacity may then be attributed to the presence of xylan in the cryogel, thus increasing hygroscopicity and improving ammonia capturing, due to ammonia being easily soluble in water [48].

**Figure 8:**
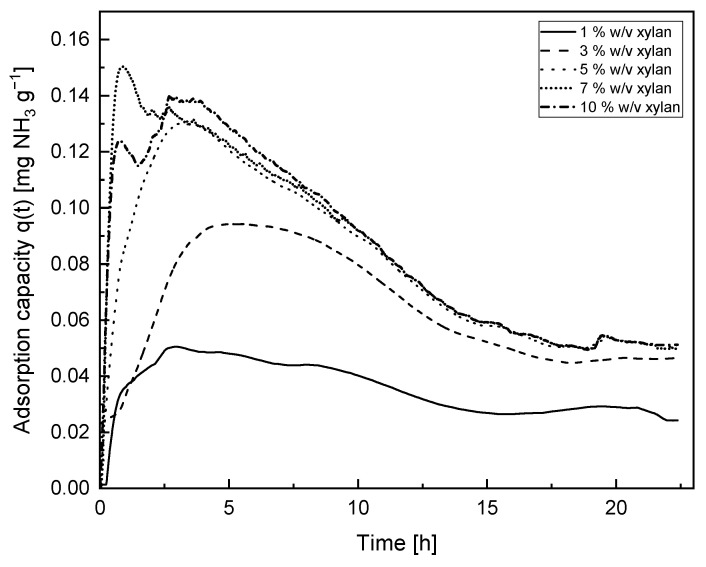
Adsorption capacity q(t) of cryogels as a function of time.

The adsorption capacity over time, calculated with Equation (9), is shown in Figure 8. The results indicate, as we mentioned before, that most of the adsorption occurs during the first 15 h, and after this time, stabilization is reached. Likewise, it is possible to observe that as the concentration of xylan in the cryogel increases, it tends to reach the same equilibrium value in the adsorption capacity, since no appreciable change is shown after 10 h of adsorption for the cryogels with 5, 7, and 10% *w*/*v* of xylan. After 24 h of adsorption, the cryogels are still capable of continuing adsorbing and the values are maintained for all the cryogels.

The kinetics of the ammonia adsorption process was evaluated according to pseudo-first- and pseudo-second-order kinetic models [36]. As it can be observed in Table 2, the experimental data correlated satisfactorily with the pseudo-second-order kinetic model. Even though both models presented an acceptable correlation coefficient (R^2^ > 0.8), the pseudo-second-order kinetic model (indicating chemisorption) showed a better fit (R^2^ > 0.95) of the data from the experimental information.

Table 3 provides a summary of the ammonia adsorption capacity of the different cryogel samples, as well as the ammonia adsorption rates and total ammonia adsorbed. As it can be observed, the cryogel sample with 10% *w*/*v* xylan exhibited the highest adsorption capacity, adsorbing 0.047 mg NH_3_/mg of the sample. It is noteworthy that the adsorption capacity increased with the increase in the xylan content of the cryogel, even though the porosity of the cryogel decreased, which is contrary to what would be expected for porous materials. This confirms that the ammonia-capturing process is driven by chemisorption, where the kinetics adjusts to a pseudo-second-order model, and that adsorption is not driven by the morphological properties of the material or physisorption.

Compared to other well-established solid adsorbent materials, the ammonia adsorption capacity of cryogels and performance under similar conditions (P = 1 atm, T = 20–27 °C) is promising. As examples, X. Long et al. (2008) [49] and T. Asada et al. (2006) [48] found a range of ammonia adsorption capacities of 0.1–10 mg NH_3_/g for activated carbons, while Khabzina and Farrusseng (2018) [50] reported higher adsorption capacities of 26–112 mg NH_3_/g for metal–organic frameworks (MOFs) and 2–56 mg NH_3_/g for zeolites.

On the other hand, in a study by C. A. Takaya et al. (2020) [2], oak biochar showed adsorption capacities lower than 3 mg NH_3_/g. Other studies report lower values between 0.01 and 0.1 mg NH_3_/g for organic materials such as ashes or other types of activated carbons. In this way, the cryogels obtained in the present study, particularly those with 5% *w*/*v* xylan, showed competitive adsorption performance, positioning them as promising eco-friendly adsorbents for ammonia removal from air pollutants.

## 4. Conclusions

This study successfully developed responsive eco-friendly cryogels from coffee residues in Colombia. The incorporation of xylan up to 10% *w*/*v* significantly enhances ammonia adsorption, reaching 0.047 mg NH_3_/g of the sample. These cryogels can be used for capturing ammonia, especially in environments with high ammonia concentrations such as poultry farms. Their preparation from agricultural waste materials constitutes an advantage when compared to other adsorbents such as MOFs, zeolites, and activated carbons due to their biological origin, availability, and cost.

## Figures and Tables

**Table 1:** BET area obtained for cryogels 1–10% *w*/*v* xylan.

Cryogel	A_BET_ (m^2^/g)	Pore Volume (cm^3^/g)
1% *w*/*v* xylan	6.0	0.0346
5% *w*/*v* xylan	2.0	0.0183
10% *w*/*v* xylan	3.0	0.0213

**Table 2:** Kinetic modeling of cryogels adsorption following pseudo-first- and pseudo-second-order equations by [33].

	Pseudo-First Order			Pseudo-Second Order		
Xylan Variation in Cryogels (% *w*/*v*)	Adsorption Capacity q_e_ (mg NH_3_/g)	k_1_ (mg NH_3_/mg _sample_ s)	R^2^	Adsorption Capacity q_e_ (mg NH_3_/g)	k_2_ (g _sample_/mg NH_3_ s)	R^2^
1	0.0523	0.000009	0.81	0.0247	0.0035	0.97
3	0.1064	0.000012	0.76	0.0427	0.0026	0.95
5	0.1376	0.000014	0.85	0.0461	0.0217	0.96
7	0.1763	0.000018	0.82	0.0460	0.0019	0.96
10	0.1522	0.000015	0.92	0.0466	0.0020	0.96

**Table 3:** Comparison of ammonia adsorption capacities reported in previous reports compared to this study.

Reference	Adsorbent	q_e_ [mg NH_3_/g]
[48]	Activated carbon	0.01–10
[49]	Activated carbon	4.8
[50]	MOFs	26–112
[50]	Zeolites	2–56
[2]	Oak biochar	<3.0
[51]	Fly ash	0.08–0.10
[52]	Activated coal	0.011
This study	Xylan cryogels (10% *w*/*v*)	0.047

## Data Availability

The raw data supporting the conclusions of this article will be made available by the authors on request.
